# Surveillance of early stage COVID-19 clusters using search query logs and mobile device-based location information

**DOI:** 10.1038/s41598-020-75771-6

**Published:** 2020-10-29

**Authors:** Shohei Hisada, Taichi Murayama, Kota Tsubouchi, Sumio Fujita, Shuntaro Yada, Shoko Wakamiya, Eiji Aramaki

**Affiliations:** 1 Nara Institute of Science and Technology (NAIST), Nara, Japan; 2 Yahoo Japan Corporation, Tokyo, Japan

**Keywords:** Epidemiology, Information technology, Public health, Epidemiology, Population screening, Infectious diseases

## Abstract

Two clusters of the coronavirus disease 2019 (COVID-19) were confirmed in Hokkaido, Japan, in February 2020. To identify these clusters, this study employed web search query logs of multiple devices and user location information from location-aware mobile devices. We anonymously identified users who used a web search engine (i.e., Yahoo! JAPAN) to search for COVID-19 or its symptoms. We regarded them as web searchers who were suspicious of their own COVID-19 infection (WSSCI). We extracted the location of WSSCI via a mobile operating system application and compared the spatio-temporal distribution of WSSCI with the actual location of the two known clusters. In the early stage of cluster development, we confirmed several WSSCI. Our approach was accurate in this stage and became biased after a public announcement of the cluster development. When other cluster-related resources, such as detailed population statistics, are not available, the proposed metric can capture hints of emerging clusters.

## Introduction

In 2019 and 2020, a disease caused by a novel coronavirus called SARS-CoV-2 spread worldwide^1^. To control the rapid spreading of this coronavirus disease 2019 (COVID-19), pandemic management interventions are important for public health authorities and relevant governmental organizations. The pandemic management interventions include optimizing arrangements of medical service supply, health and medical information dissemination, and control and development of relevant rules and regulations. Among various interventions, the cluster response, which intends to detect small groups of infected people in a large community, is significant in the early stages of a pandemic, as this enables health authorities to restrict the spread of the virus^2^.

On February 25, 2020, the Ministry of Health, Labour, and Welfare (MHLW) of Japan convened a team of about 30 specialists to identify clusters; by March 17, 2020 they had identified 13 clusters^3^. There were possibly more clusters since young people, who tend to have mild symptoms compared to the elderly^4^, actively commuted to work or to school. Considering the business and social activities of youth, it is crucial to capture the slight signals of the infection in them.

This context motivated us to leverage the usage logs of multiple devices (smartphones, tablets, and PCs) through which we could collect location information from users who used an application on location-aware mobile devices (i.e., smartphones and tablets), anytime and anywhere. First, we assumed that a person who might have COVID-19 would attempt to obtain detailed information on the condition and its symptoms through a web search. Our assumption originates from the study^5^ that utilizes influenza-related web search queries, such as “cold” and “fever” . Ginsberg et al. reported that the aggregation of these queries highly correlated with the number of influenza patients. In the same way, we estimated the number of COVID-19 patients. Considering this, we designed 63 query patterns, such as “likely to be corona”, “cough” and “corona”, “diarrhea” and “new type”, and “coughing up phlegm” and “new type pneumonia”. We collected all available search log data from smartphones, tablets, and PCs. We defined web users whose queries were related to the symptoms of COVID-19 as web searchers who were suspicious of their own COVID-19 infection (WSSCI). We then selectively extracted location information of WSSCI from the “Yahoo! JAPAN App”. The application is one of the most popular mobile operating system applications in Japan that runs on iOS and Android OS and hosts many services such as web search and weather reports. The location data were collected from users who used the application on location-aware mobile devices (i.e., smartphones and tablets) and approved use of their location information for the research purpose. Subsequently, we counted the number of WSSCI in each day and each area based on their location information.

A previous study^5^ demonstrated that symptom-related search queries have an advantage of capturing early signals of infectious diseases. In addition, some studies^6–8^ attempted to utilize search queries for forecasting or predicting influenza epidemic. A recent study^9^ reports that both search queries and social network data can produce precise and usable estimation of the influenza development by investigating which kind of data source leads to better results. A recent work-in-progress paper also uses web search queries to predict the country-level COVID-19 epidemic^10^. To evaluate the transferability of the prediction model, these authors investigated the Italian prediction model in the other seven countries without having experienced any of them yet. In contrast, we utilized web search query logs *per user* to detect WSSCI and gather their location histories to identify locations they visited or passed through, resulting in COVID-19 *cluster* detection.

We believe that our approach is suitable for COVID-19 cluster detection because smartphone applications are widely used nowadays. This study investigates the feasibility of our approach through case studies of the COVID-19 clusters that occurred in February 2020, in Hokkaido, Japan. The COVID-19 pandemic indeed made us realize that obtaining reliable information on the current status during a pandemic crisis is challenging. However, even in such a low resource condition, smartphone users are still available and can be regarded as a type of *social sensors* who voluntarily report current events in real time,in most cases, without realizing it. To take advantage of social sensors, it is essential to examine the validity of the WSSCI-based approach in advance.

## Results

**Figure 1:**
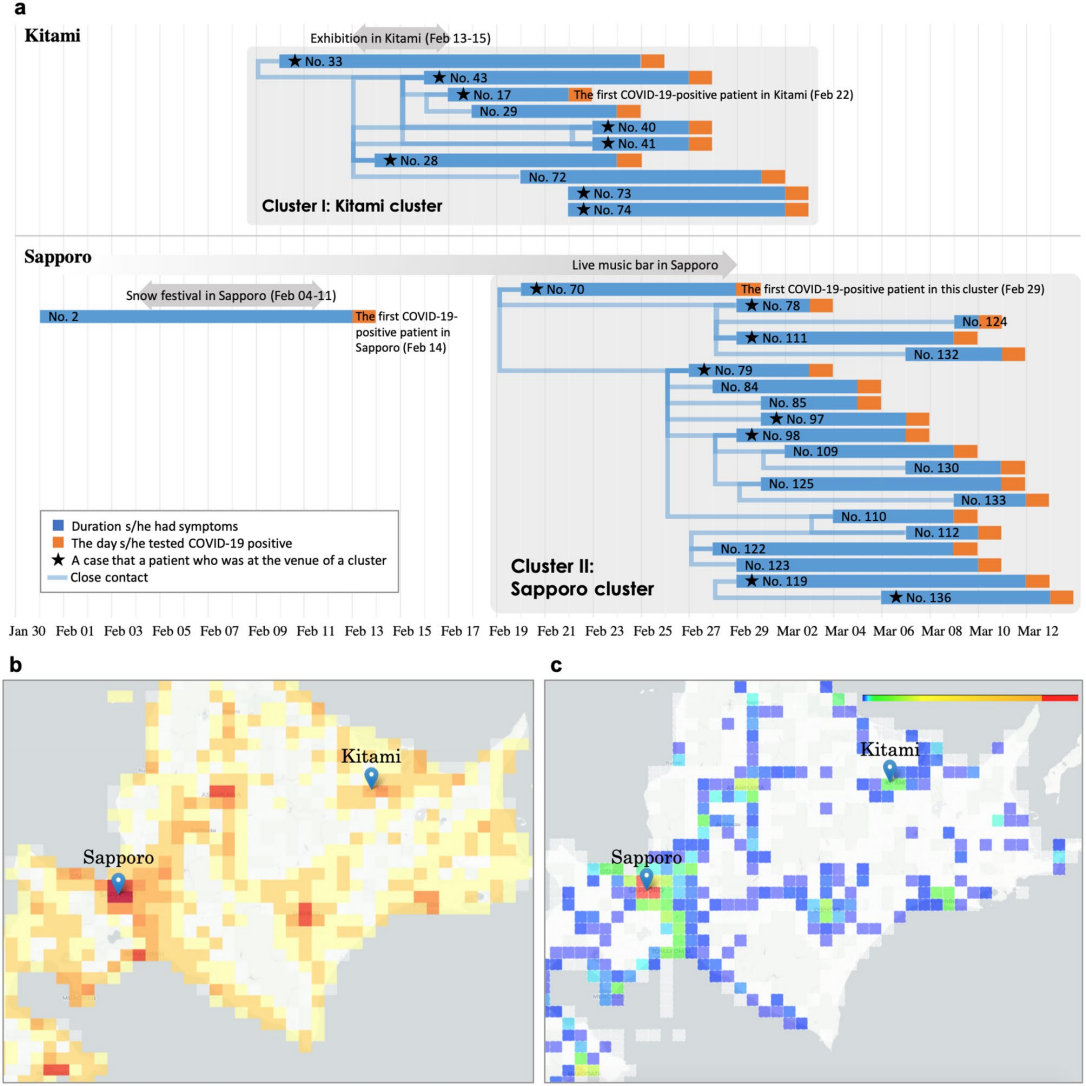
Occurrence status of the two clusters in Hokkaido, spatial distribution of population, and the total number of WSSCI per half grid (WSSCIphg) in Hokkaido. (**a**) Timeline of outbreaks of two clusters in Hokkaido, Cluster I: Kitami cluster and Cluster II: Sapporo cluster, based on the Hokkaido government’s official announcement. The existence of the Kitami cluster and Sapporo cluster was admitted on March 9 and March 18, 2020, respectively. (**b**) Population distribution in Hokkaido. The color scale ranges from red (large) to yellow (small) and white (zero). The blue marker denotes the location of a cluster (Kitami cluster is [43.804878, 143.897225] and Sapporo cluster is [43.068700, 141.350767]). (**c**) Spatial distribution of the total number of WSSCIphg throughout every 400 half grid squares between January 27 and March 1, 2020. The colors of grid squares represent the total WSSCIphg throughout every 400 half grid squares, of which red is large, blue is small, and white is zero. The blue marker denotes the location of a cluster (Kitami cluster is [43.804878, 143.897225] and Sapporo cluster is [43.068700, 141.350767]).

We focused on the area of Hokkaido where two COVID-19 clusters were reported in March 2020, according to cluster maps^3,11^ released by the MHLW. Figure 1a shows the timelines of the clusters based on official announcements by the Hokkaido government^12^, Kitami cluster^13^ and Sapporo cluster^14^. Figure 1b shows the spatial distribution of population in Hokkaido. Figure 1c shows the total number of WSSCI per half grid (WSSCIphg) between January 27 and March 1, 2020 in Hokkaido.

**Figure 2:**
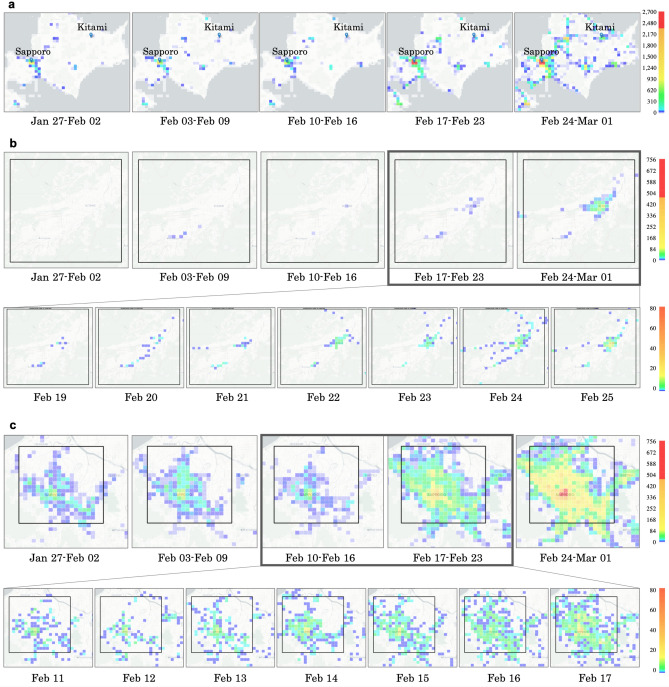
Spatio-temporal distribution of the number of WSSCI per half grid (WSSCIphg) in Hokkaido and in the target areas, Kitami and Sapporo. The colors of grid squares represent the total WSSCIphg throughout every several half grid squares, of which red is large, blue is small, and white is zero. (**a**) The distribution of total weekly WSSCIphg throughout every 400 half grid squares in Hokkaido, Japan. The blue marker denotes the location of a cluster (Kitami cluster is [43.804878, 143.897225] and Sapporo cluster is [43.068700, 141.350767]). (**b**,**c**) [Top] The distribution of total weekly WSSCIphg throughout every four half grid squares in Kitami and Sapporo, respectively, surrounded by a rectangle. [Bottom] The distribution of total daily WSSCIphg throughout every four half grid squares between 02/19 and 02/25 in Kitami surrounded by a rectangle and between 02/11 and 02/17 in Sapporo surrounded by a rectangle, respectively. The figure shows 3 days before and 3 days after the announcement of the first patient who was positive in each city (over a total of 7 days).

Figure 2a–c show the spatio-temporal distributions of WSSCI in the areas of Hokkaido, Kitami, and Sapporo, respectively. In Fig. 2a, WSSCI hot spots existed in the two cluster areas before they were reported, suggesting that WSSCI could be a clue for the cluster build-up. However, there is no cluster in several WSSCI hot spots near the central and the southern part of Hokkaido. This suggests that not all hot spots are clusters, exposing the limitation of our approach.

**Figure 3:**
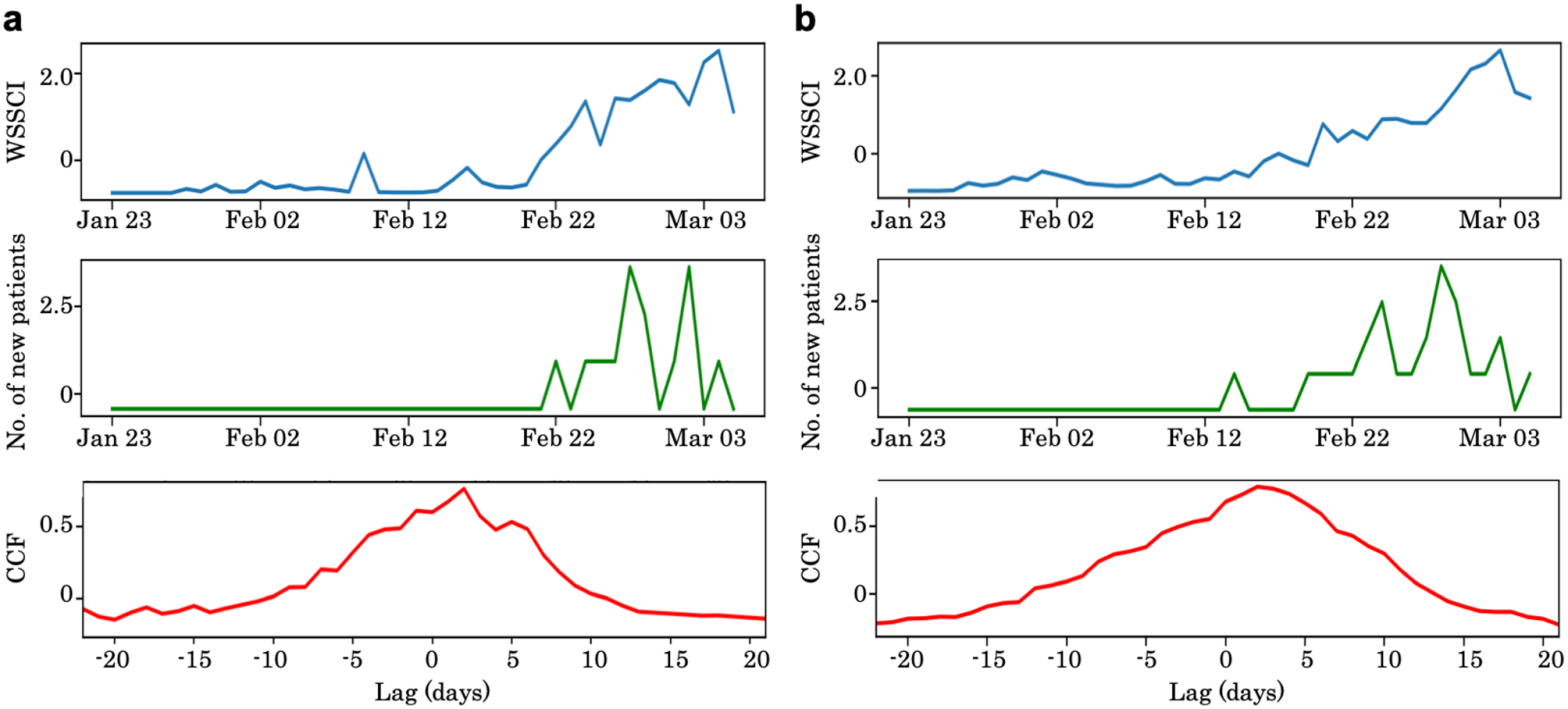
Cross-correlation function (CCF) between time series of WSSCI and the number of new patients in the subprefectures including the target areas (**a**) Kitami and (**b**) Sapporo. [Top] Time series of the WSSCI (standardized). The x-axis indicates the timeline. [Middle] Time series of the number of new patients (standardized). The x-axis indicates the timeline. [Bottom] CCF between the two-time series. The x-axis indicates lag (0 is the highest correlation day). The y-axis indicates correlation. In (**a**) Kitami, its CCF is 0.766, whose lag is 2 days. In (**b**) Sapporo, the CCF value is 0.786, whose lag is 2 days. Both show a significant correlation (p< 0.05).

To check the correlation between WSSCI and the number of new patients in the areas of Hokkaido, Kitami, and Sapporo, respectively, the cross-correlation (CCF), mentioned in “Methods” section, was calculated. As a result, in Hokkaido as a whole, the CCF between WSSCI and the number of new patients showed 0.803, with a time lag of 2 days. The CCF informed us about temporal trends in the areas where the clusters occurred as shown in Fig. 3. In the Kitami area (Fig. 3a), the WSSCI has a maximum cross-correlation of 0.766, determined 2 days after the change in the number of new patients. In the Sapporo area (Fig. 3b), the WSSCI has a maximum cross-correlation of 0.786, estimated 2 days after the change in the number of new patients. These results suggest that the WSSCI have also increased in response to the increase in the number of new patients in each area.

### Cluster I: Kitami cluster

The first cluster comprises more than 10 cases related to the participants of an exhibition and a dinner party in Kitami between February 13 and 15, 2020. Eight participants have been confirmed to be infected in this event, as shown in the upper part of Fig. 1a. Starting with the case reported in Kitami on February 22 (No. 17), five participants (Nos. 17, 33, 43, 73, and 74) including the first case in Kitami and three participants from Sapporo (Nos. 28, 40, and 41) have been confirmed to be infected. Regarding the interpersonal relations, person No. 29 was in close contact with person No. 17, and person No. 72 with person No. 28.

Before the breaking news of the first patient infected with COVID-19 in Kitami on February 22, 2020, we could confirm several WSSCI between February 3 and 16, 2020, in Fig. 2b, which were not confirmed at all in the previous week, January 27 to February 2, 2020. After the breaking news on February 22, the WSSCI spread across this area. From this fact, it could be inferred that the appearance of WSSCI might indicate the cluster existence, demonstrating the feasibility of the WSSCI-based cluster prediction. The lower part of Fig. 2b shows the spatio-temporal distribution of WSSCI; we can see some hot spots of WSSCI on February 19, 2020. Considering the initial status of this area between January 27 and February 2, 2020, that is the nonexistence of WSSCI, as shown in Fig. 2b, these hot spots might provide us hints about the cluster existence or the possibility of cluster occurrence in the near future.

### Cluster II: Sapporo cluster

In another cluster in Hokkaido, as shown in the lower part of Fig. 1a, eight people have been confirmed to be infected in a live music bar in Sapporo. Starting with a store clerk, No. 70, who was tested positive on February 29, 2020, after visiting a medical office, other two clerks (Nos. 78 and 79) were also positive on March 3, 2020. The bar remained open until February 29, 2020, when the first person tested positive. Two guests (Nos. 119 and 136) visited the bar on February 25, 2020, one from Otaru and the other from Kitami. The other three guests (Nos. 97, 98, and 111) who visited the bar on February 26, 2020, have been confirmed to be infected also. In addition, the infection spread to a total of 12 close contacts of the people who had been in the live music bar.

In Sapporo, the cluster occurrence was first reported on March 17, 2020^3^. Unlike the Kitami cluster, the first patient who tested positive in Sapporo was not directly related to the Sapporo cluster. Furthermore, there were already 13 patients who tested positive in Sapporo, and 66 patients tested positive in the entire Hokkaido before February 29, 2020, when the first case of the Sapporo cluster was confirmed. Therefore, although we can confirm that WSSCI clearly increased and spread across Sapporo during the week when the first positive case of Sapporo cluster (February 24–March 1, 2020) was confirmed, many WSSCI were already spreading across the area before the week of February 10–16, as shown in Fig. 2c.

To investigate more detailed trends of WSSCI, the lower part of Fig. 2c shows the daily spatial distribution of WSSCI in Sapporo from February 11 to February 17, 2020. On February 11, 2020, several WSSCI had already existed, indicating that this area is a part of densely populated city in terms of both population statistics as shown in Fig. 1b and WSSCI as shown in Fig. 1c. On February 14, 2020, the occurrence of the first case of a suspected patient in this area made national headlines. However, there was no cluster at that time yet (unlike Cluster I: Kitami cluster). In such a situation, many WSSCI had appeared after the report of the suspected patient on February 14, 2020. The reason for this sudden burst of WSSCI is that many people attempted to obtain more information about COVID-19 even though they did not have any symptoms. This indicates that the WSSCI-based approach could be easily biased by media coverage.

## Discussion

### Advantages

This study attempted to detect early clusters by identifying people who suspected their own COVID-19 infection using web search query logs and by extracting their location information. The WSSCI-based approach is capable of identifying potential COVID-19-positive patients using web search query logs. Although statistics of inpatients and outpatients have been reported, COVID-19 patients with mild symptoms who do not go to the hospital are difficult to identify. In fact, even patients with mild symptoms can transmit the virus to others; it is therefore important to identify these patients. Fortunately, our approach can identify such patients through their location information.

An advantage of the WSSCI-based approach is the real time nature of the users. In reality, it is difficult to follow up the population size accurately, as people can move from one city to another to avoid COVID-19. For example, remote-working enables people to work from anywhere, so that some move to less populated areas and others go back to their hometowns and live with their parents^15,16^. It might cause a dynamic change of population distribution as shown in Fig. 2c. Even in such a situation, the WSSCI-based approach can capture ongoing local migrations. Additionally, we realized that it might be effective to support a real-time cluster surveillance in developing countries, where we can safely assume at least the existence of web search traffic in comparison to other smartphone applications (e.g., location-sharing social network services).

### Limitations and future direction

This study on the ongoing pandemic crisis has the advantages described above, but there are limitations that should be considered to apply syndromic surveillance. One limitation is that an area with high concentration of WSSCI does not always indicate the existence of a cluster. In fact, among several hot spots in Fig. 1B, the number of clusters officially detected was only two, causing an overestimation of clusters. However, as mentioned above, it would be helpful to narrow down potential areas that might be clusters in the early stages.

Another limitation is a bias caused by media coverage. Whenever any mass media report the location of a COVID-19-positive patient, many people who are in areas adjacent to the reported location submit COVID-19-related queries to acquire additional information. Therefore, media could change the nature of WSSCI, suggesting that our approach would be more effective to detect potential cluster areas before any national headlines.

We also investigated another factor, news media, represented by the number of news articles that may affect WSSCI. The CCF between WSSCI and the number of news articles showed the highest CCF = 0.902, whose lag is 0 days; the CCF between the number of patients and the number of news articles also presented a similar result that the CCF is 0.793, whose lag is 0 days. In other words, the number of news articles rapidly reflects with the patient number, but it is not that accurate (0.793). In contrast, there is a lag of 2 days between WSSCI and the number of new patients, but it has a similar correlation (0.803). Here, one important question arises as to whether the WSSCI increase was caused by mass media reports or the occurrence of news about the patients. Based on this result alone, however, we could not answer the question. Although we could not determine the actual causal relations among the three factors, we could conclude that the WSSCI are highly correlated with the previous (2 days before) patient number. Investigating the news biases to WSSCI is one of the remaining future tasks.

The cross-correlation analysis indicates the difficulty of the WSSCI-based real-time cluster detection because there is a lag of 2 days. Strong news bias made it hard to observe the precise effect of WSSCI. Still, several practical applications are possible because the WSSCI detected the increase in the number of new patients 2 days later. Even if it would be challenging to develop a nation-wide surveillance system, which would be rarely required at the early stage of this crisis, a government could detect the patient increase in any local cities, leading to rapid local support, including the arrangement of medical facilities, specialist management, and so on.

## Methods

### Material

We utilized web search query log data and location information provided by Yahoo Japan Corporation^17^ as signals for syndromic surveillance. Yahoo Japan Corporation hosts a wide variety of over 100 services of wide variety, including web search, news, maps, weather forecasts, online shopping, and knowledge search on a famous portal site called the “Yahoo! JAPAN” and a popular mobile operating system application called the “Yahoo! JAPAN App”. According to the report^18^, these services are used among a wide range of users of all ages, gender, areas, occupations, and annual income, with little difference based on any of these categories. The number of monthly active users is approximately 82.6 million (61.8 million users on smartphones, which is about 90% of the smartphone users in Japan and 20.8 million users on PCs, which is about 70% of the PC users in Japan) and its market share is second to that of Google^19^.

The Yahoo! JAPAN search engine stores more than 10 billion search queries on all devices to their server. We extracted search query logs related to COVID-19 for the identification of infection suspecting searchers as described in the following subsection. More than one million of all “Yahoo! JAPAN App” users who explicitly approved the use of their mobile device location information for research purposes, constituted the study sample. This App stores the GPS location information to the server. Due to the privacy policy of Yahoo Japan Corporation, we could not utilize data that could identify an individual user. Instead of individual tracking, we investigated area-based statistics data.

### Infection suspecting searcher identification using web search query logs

We leveraged the search logs on all devices from Yahoo! Japan Search^20^ to identify search users who were possibly affected by the disease and were looking for information about the characteristic features of its symptoms to check whether their symptoms corresponded to typical cases of the disease.

Queries submitted by search users were matched against 63 predefined patterns, consisting of three single-term patterns and 60 double-term patterns. Single-term patterns included Japanese short phrases, literally meaning “likely to be corona”, “likely to be corona-virus”, or “likely to be new type pneumonia”. Double-term patterns included one of three main terms succeeded by a facet term out of 20 patterns. The main terms were “corona%”, “new type”, or “new type pneumonia”, where $\%$ denoted wild card matching, as used in the “like” operator of the SQL language. The facet terms included 18 Japanese phrases meaning one of the symptoms, namely, “cough”, “diarrhea”, “coughing up phlegm”, “slight fever”, “headache”, “cold”, “fevered”, “no fever”, “without fever”, “high fever”, “develop fever”, “runny nose”, “chills”, “throat”, “chest”, “phlegm”, and “feel tired” or “weariness”, or two phrases meaning “designated hospitals” (which means hospitals validated by the local health authorities as specialized for treatments of COVID-19-infected patients) or “advice” (which was presumably intended for special consultation services in charge of advising those who suspected to be infected with the virus). These terms in double patterns were AND-combined; therefore, their order was irrelevant. A query was matched against a pattern if and only if all terms were matched against any terms in user queries. Therefore, for queries matched against one of the 63 aforementioned patterns, anonymized user IDs of searchers were stored only when they had already approved the use of their smartphone location information for research purposes. These searchers were defined as web searchers who are suspicious of their own COVID-19 infection (WSSCI).

### User location information extraction

Among the app users who explicitly approved the use of their location information, we mapped the number of WSSCI using the symptom suspected queries described in the previous subsection into each half grid square (500 m $\times$ 500 m) according to the location information. Concerning the privacy, we used neither the IDs of each searcher nor their exact location information, but only the number of searchers in each day in the areas defined by the half grid square code system^21^, which we called the number of WSSCI per half grid (WSSCIphg). The number of searchers was counted only when the location information of the searchers was stored during their stay in the grid area.

### Cross-correlation

To determine whether the WSSCI could predict clusters or not, we calculated the cross-correlation function (CCF) between the WSSCI and the number of new patients for each area, Sapporo and Kitami. To investigate the relationship between WSSCI and the number of new patients, we also examined the cross-correlation and lag between them, which indicate a hint for their causal relations. To investigate a bias caused by the news broadcast, we examined the relationship between WSSCI, the number of new patients, and the number of news articles related to COVID-19 in the target area, Hokkaido. To do so, we used news articles that contain at least one of the keywords “coronavirus” or “pneumonia” from a news article archive site^22^. Given two time series of data, the CCF identifies a similarity and chronological difference between them by incrementally shifting one time series vector and repeatedly calculating the correlation between two signals. A chronological difference is called a lag, which represents how many days apart the two time series are. If the peak correlation is at the center (lag = 0), this indicates that the two time series are most synchronized at that time. To compute the CCF, we used one of the Numpy modules, numpy.correlate^23^.

### Ethics statement

All participants provided written (or electronically displayed) informed consent before participating in this study and agreed to the terms and conditions of the “use of services” provided by Yahoo! Japan services when they disclosed their location. This study did not require the participants to be involved in any physical and/or mental intervention. Participants’ information was unlinkable, anonymized, and deidentified prior to analysis. This research did not obtain identifiable private information, meaning that it was exempt from Institutional Review Board approval according to the Ethical Guidelines for Research of the Japanese national government. Yahoo Japan Corporation has approved the use of the participant data in this study.

## Data Availability

Although the data that support the findings of this study are not allowed to be publicly available according to the privacy policy and data disclosure policy of Yahoo Japan Corporation and the Japanese privacy law, the corresponding author can comply with a reasonable request.

## Code availability

The custom codes in these analyses are available^24^.
